# A Histopathological Comparison of Pulpal Response to Chitra-CPC and Formocresol used as Pulpotomy Agents in Primary Teeth: A Clinical Trial

**DOI:** 10.5005/jp-journals-10005-1126

**Published:** 2012-02-24

**Authors:** N Ratnakumari, Bijimol Thomas

**Affiliations:** Vice Principal, Professor and Head, Department of Pedodontics Government Dental College, Kottayam, Kerala, India, e-mail: drretnasarnga@rediffmail.com; Senior Resident, Department of Pedodontics, Government Dental College, Kottayam, Kerala, India

**Keywords:** Pulpotomy agent, Sree Chitra-Calcium Phosphate Cement, Formocresol

## Abstract

Preventive measures have helped to minimize the occurrence of dental caries. However, premature loss of primary teeth on account of dental caries still remains a common problem among children. The pulpotomy technique has been the choice for treating vital primary and young permanent teeth with carious, mechanical and traumatic pulp exposures. The ideal pulpotomy medicament should be bioinductive or at least biocompatible, bactericidal and harmless to the pulp and surrounding structures. It should also promote healing of the radicular pulp and prevent bacterial microleakage with the least interference in the physiological process of root resorption. Since the best criteria for judging the effectiveness of a medicament when used for vital pulp therapy is the response that it produces in the pulp**.** The purpose of the present study was to evaluate and compare the response of human pulp tissue to recently developed Indian material, Sree Chitra-Calcium Phosphate Cement (Chitra-CPC) and formocresol, used as pulpotomy agent in deciduous teeth. Chitra-CPC has been compared with formocresol, taking into account that formocresol is still considered the gold standard in primary tooth pulpotomy. The study was conducted among 10 children in the age group of 8 to 12 years focusing on 20 noncarious primary canines indicated for serial extraction. Each patient received two different pulpotomy procedures—one in each of the primary canines using formocresol and the other with Chitra-CPC as pulpotomy agents. After 70 days, the teeth were extracted and subjected to histological examination. The results did not reveal statistically significant difference between the two groups. But Chitra-CPC gave more favorable results, in respect of pulpal inflammation, dentin bridge formation, quality of dentin bridge and connective tissue in dentin bridge.

**How to cite this article:** Ratnakumari N, Thomas B. A Histopathological Comparison of Pulpal Response to Chitra- CPC and Formocresol used as Pulpotomy Agents in Primary Teeth: A Clinical Trial. Int J Clin Pediatr Dent 2012;5(1):6-13.

## INTRODUCTION

The ideal pulpotomy material or drug should be bioinductive or at least biocompatible, preserve healthy radicular pulp tissue, be bactericidal, be harmless to the pulp and surrounding structures, promote healing of the radicular pulp, prevent bacterial microleakage and should not interfere with the physiological process of root resorption.^10^Formocresol is the most commonly used pulpotomy agent in deciduous teeth. It is a formaldehyde-cresol mixture and the rationale for its use is its bactericidal and pulpal fixative properties. Clinical and radiographic studies have shown that formocresol pulpotomies have success rates ranging between 70 and 97% (Fuks and Bimstein, 1981).^2^ Yet, regardless of its favorable clinical results, formocresol has come under close scrutiny because of the concerns regarding the systemic toxicity, immune response, mutagenic and carcinogenic potential embryotoxicity, and teratogenicity^8^and defects in succcedaneous teeth (Messer et al, 1980; Zarzar et al, 2003). The International Agency for Research on Cancer (IARC) classified formaldehyde as carcinogenic to humans in June 2004, leaving the profession to look for other viable alternatives to formocresol.^12,18^

Several medicaments have been substituted for formocresol with varying rates of success. Glutaraldehyde was proposed as a pulp tissue fixative by Gravenmade in 1975. Advantages of this include nonreversible fixation and self-limiting penetration. With the similar toxic effects to formocresol, and absence of strong evidence of improved success rates, glutaraldehyde was not accepted as an alternative to formocresol. Ferric sulfate was used due to its hemostatic effect.^9^ Although the metal protein clot at the surface of the pulp stump acts as a barrier to the irritating components at the subbase, it functions solely in a passive manner and its use is arguably technique-sensitive.^11^Calcium hydroxide, a regenerative pulpotomy agent, was reported to be a failure in primary teeth due to higher incidence of the development of chronic pulpal inflammation and internal resorption. Mineral trioxide aggregate (MTA) pulpotomy has recently attracted attention because of its excellent sealing ability, biocompatibility, ability to stimulate hard tissue formation and higher long- term clinical and radiographic success rate. Of late, a drawback to the clinical use of MTA was observed related to its cost and perceived problems with the storage.^20^ Other alternatives, such as electrosurgery, different laser irradiating systems like Nd:YAG, Er:YAG, and carbon dioxide, bone morphogenic proteins, collagen, enamel matrix derivative and freeze-dried bone were also found to be not ideal for pulpotomy.

In the beginning of 21st century, with the greater understanding of the pulp biology, pathophysiology and its power of healing and with the materials that are not only biocompatible but also bioinductive, the emphasis was shifted from devitalization to regeneration of the remaining pulp tissue. One such material, which has shown immense potential for regeneration is calcium phosphate biomaterials. Calcium phosphate cement (CPC), a member of calcium phosphate biomaterials, is considered as a new generation bone substitute with potential clinical applications in orthopedics and dentistry. The cement mass undergoes isothermal setting and gets converted into hydroxyapatite, the basic inorganic component of bone and teeth. The researchers have projected several merits for CPCs as a dental material. CPCs are identified as the most suitable sealer/filler materials in endodontic treatment, for augmenting periodontal defects and as bone filler for gaps around oral implants. CPCs have been proved to be useful for direct pulp capping in animal studies.^4,19^

‘Chitra-CPC’ is a new CPC formulation with good rheological property. This material was developed in India at the Sree Chitra Tirunal Institute for Medical Science and Technology (SCTIMST), Thiruvananthapuram. Evaluation of the safety and efficacy of the cement is done according to the national and international standards of guidelines. Since it is biocompatible, osteoconductive, moldable, nontoxic, nonimmunogenic with good sealing ability with the least mutagenic or carcinogenic potential. Chitra-CPC apparently satisfies all the requirements of an ideal pulpotomy material.^7,15^ Animal studies have already proved the efficacy of CPC as a pulp dressing material. As human studies have not been conducted on the same, the present study has brought about the baseline for further studies in evaluating Chitra-CPC as a promising pulpotomy material for human primary teeth.

The present study was undertaken with a view to evaluating the efficacy of Chitra-CPC as a pulpotomy agent in comparison with formocresol, the most commonly used agent through histopathologic responses of pulpal tissues of human deciduous teeth.

## METHODOLOGY

### Study Population

Children in the age group of 8 to 12 years with primary canines indicated for serial extraction at the Department of Pedodontics, Government Dental College, Thiruvananthapuram, constituted the study population. Clearance of Ethics Committee was obtained initially. Subsequently, informed consent of parents and patients was obtained before proceeding with the study. The sample for the study constituted 20 noncarious primary canines of 10 children.

### Inclusion Criteria

It was also ensured that the patients were in good general health and free of any systemic disease including congenital heart diseases and bleeding disorders and the teeth included in the study should be free of caries, hypoplastic defects or any malformations. It was also made sure that there were no history of pain/tenderness to percussion; control of hemorrhage was possible at the exposure site; no apparent mobility to finger pressure; and teeth are free of any periodontal problems for the study group.

## MATERIALS

### Chitra-Calcium Phosphate Cement (Chitra-CPC)

Prepared by the Sree Chitra Tirunal Institute for Medical Sciences and Technology (SCTIMST), Thiruvananthapuram. The cement has been tested for safety and efficacy and approved for human clinical use by the Institutional Ethics Committee.

### Formocresol

Formocresol used in the present study was Trisol, marketed by Vishal Dentocare Pvt Ltd.

## PROCEDURE

From among the study population, 20 noncarious deciduous canines (both left and right) either from maxillary or mandibular arch satisfying the inclusion criteria were selected. Each patient received two different pulpotomy procedures in the primary canines to be extracted. For this, selected teeth in each patient were divided into two groups— Group A (left canines) and Group B (right canines). Formocresol was used as pulpotomy agent for Group A and Chitra-CPC as pulpotomy agent for Group B.

All the teeth in both treatment groups were observed to be clinically successful at each follow-up visit. In addition, all the patients were found to be free of pain subsequent to the treatment, and clinical signs or symptoms of infection were not observed in any of the patient covered under the study. Teeth from each group were extracted in 70 ± 5 days after administration of local anesthesia, for evaluation of pulpal response. After extraction, about 2 mm of the apices of the teeth were cutoff to facilitate fixation of the pulp tissue. These specimens were fixed in 10% formalin for 48 hours.

### Tissue Processing

After fixation, teeth were dehydrated in ascending grades of alcohol at room temperature, immersed in alcoholic acetone (1:1 v/v) mixture followed by immersion in one change of 100% alcohol. The teeth were kept in two changes of washed monomer and embedded in PMMA. Thin sagittal sections (100 to 150 μ m) of resin blocks were cut using a high-precision diamond saw (ISOMET 5000, Beuhler). The sections were polished using a variable speed grinder- polisher (ECOMET 3000, Beuhler) and subsequently stained with Stevenel’s blue and hematoxylin and eosin. The stained sections were examined under stereomicroscope (LEICA, Germany) and trinocular transmitted light microscope (NIKON, Japan) and images were captured using a digital camera.

### Outcome of Histopathologic Assessment

The sections were blindly evaluated by a histopathologist (Sree Chitra Tirunal Institute for Medical Sciences and Technology, Thiruvananthapuram) using the following criteria (modified scoring system adapted from Stanley^3^ as indicated in Tables 1 to 8.

## STATISTICAL ANALYSIS

The data collected from the histological examinations were statistically analyzed using the Chi-square and the Mann- Whitney U-test for differences between the control formocresol groups and the experimental Chitra-CPC groups according to the grading criteria. The level of significance was set at a p-value of less than 0.05.

**Table 1:** **Table 1: **Degree of pulpal inflammation

*Score*		*Description*	
0		No inflammation	
1		Mild inflammation infiltrate	
2		Moderate inflammation infiltrate	
3		Heavy inflammation infiltrate	
4		Abscess	

**Table 2:** **Table 2: **Tissue reaction to the material

*Score*		*Description*	
0		No macrophages/giant cells adjacent to the material	
1		Mild infiltration of macrophages/giant cells	
2		Moderate infiltration of macrophages/giant cells	
3		Severe infiltration of macrophages/giant cells	

**Table 3:** **Table 3: **Impaction of particle of pulp capping agent

*Score*		*Description*	
1		No impaction of pulp capping agent	
2		Impaction of pulp capping agent	

**Table 4:** **Table 4: **Presence of dentin chips

*Score*		*Description*	
0		No dentin chips	
1		Small dentin chips	
2		Double dentin bridges	
3		Pulp stones	

**Table 5:** **Table 5: **Dentin bridge formation

*Score*		*Description*	
0		No presence of dentin bridge formation	
1		Bridge formation <25%	
2		Bridge formation >25% but <50%	
3		Bridge formation >50% but <75%	
4		Bridge formation >75%	

**Table 6:** **Table 6: **Location of dentin bridge

*Score*		*Description*	
0		No presence of dentin bridge formation	
1		At the interface of exposure of pulp	
2		Not at the interface of exposure of pulp	
3		Combination	

**Table 7:** **Table 7: **Quality of dentin formation in bridge

*Score*		*Description*	
0		No presence of dentin bridge formation	
1		No tubules present	
2		Irregular pattern of tubules	
3		Regular pattern of tubules	

**Table 8:** **Table 8: **Connective tissue in dentin bridge

*Score*		*Description*	
0		No presence of dentin bridge formation	
1		No connective tissue	
2		CT <25%	
3		CT >25% but <50%	
4		CT >50% but <75%	
5		CT>75%	

CT: Connective tissue

## RESULTS

In all the samples, the capping agent was identified as a granular grey material and the sealer was identified as a homogenous grey to blackish material.

### Formocresol

**Fig. 1:**
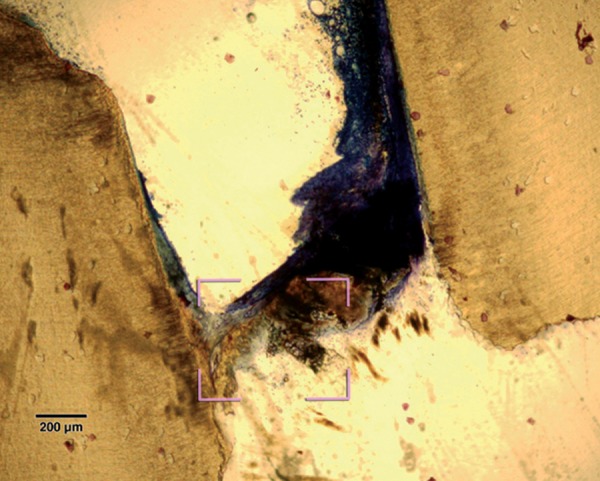
A poorly calcified dentin bridge formation in the sample treated with formocresol (Stevenel's Blue staining, at 4× mag)

Pulp tissue subjacent to the exposure site showed a layer of dense homogenous eosinophilic tissue. Heavy inflammation was present with neutrophils, lymphocytes, and foamy macrophages in six samples as seen in the figures. One sample had a score of 4 indicating abscess formation. Area apical to the inflammatory zone appeared normal, i.e. healthy, vital pulp tissue in most of the samples. Layers of odontoblasts adjacent to the capping agent were disorganized. Two samples presented poorly calcified dentin bridge formation at the apical third of the root canal shown in Figure 1. Dentin chips were observed in seven samples. None of the samples showed tissue reaction to the material or impaction of material into the pulp.

### Chitra-CPC

Pulp tissue below the capping material showed varying degrees of inflammation. Four samples showed no inflammation. Moderate inflammation was present in two samples as seen in the respective figures. Remaining four samples had heavy inflammation with neutrophils and foamy macrophages. The area apical to the inflammatory zone appeared normal with healthy, vital pulp tissue (Figs 2A and B). The layer of odontoblasts adjacent to the capping agent was disorganized. Patchy areas of mineralizing reparative dentin were noted along the dentin wall in most of the samples. Six samples, showed dentin bridge formation. In respect of three samples, dentin bridge formed at the exposure site (Figs 3A to 4) and in three just below the exposure site (Figs 2A and B). In four samples, dentin bridge formation was complete or nearing completion (Figs 2A to 3B). Two samples showed partial dentin bridge formation (Fig. 4). Three samples presented regular dentin tubules in the bridge. In most of the samples, dentin bridge did not contain connective tissue. Dentin chips were observed in six samples. None of the samples showed impaction of capping agent.

On grading the sections according to the criteria that were based on a modified scoring system adapted from Stanley, scores and observations after 70 days are given in Table 9.

It was found that there was no statistically significant difference between the formocresol and the Chitra-CPC group in any of the criteria. But Chitra-CPC gave more favorable results with regard to the pulpal inflammation (mean score of 1.6 for Chitra-CPC, 2.6 for formocresol, p-value 0.143), dentin bridge formation (mean score of 2 for Chitra-CPC, 0.80 for formocresol, p-value 0.190), quality of dentin bridge (mean score 1.4 for Chitra-CPC, 0.2 for formocresol, p-value 0.075) and connective tissue in dentin bridge (mean score 1.2 for Chitra-CPC, 1.0 for formocresol, p-value 0.280).

## DISCUSSION

When the pulp tissues from primary teeth, observed clinically successful after pulp therapy, were evaluated histologically the number of samples classed as successful did reduced.^17^ Since the best criteria for judging the effectiveness of a medicament when used for vital pulp therapy is the response it produces in the pulp, the present study evaluated the efficacy of Chitra-CPC as a pulpotomy agent in human deciduous teeth through histopathologic evaluation of pulpal response. It was compared with formocresol, taking into account that formocresol is still considered the gold standard in primary tooth pulpotomy and the tissue reactions of the dental pulp to formocresol were well examined histologically in many studies.^1,6,14^Chitra-CPC was used in the present study as a pulp dressing material for primary teeth because it was highly biocompatible for its composition, almost identical to that of tooth and bone mineral and it encouraged hard tissue regeneration.^7,19^

In this study, the most common histologic feature of pulp tissue fixed with formocresol was that subadjacent to the formocresol was a dense layer of eosinophilic fixed zone below which was the inflammatory zone with neutrophils, macrophages and lymphocytes (Figs 5A to 6B) followed by the normal healthy vital pulp tissue in majority of samples. These findings were corroborative with the findings of various studies.^5^ After Chitra-CPC pulpotomy, the pulp tissue showed varying degrees of inflammation without initial necrosis (Figs 7A to 8) followed by normal healthy vital pulp similar to the studies by Jean et al (1998).

Current study demonstrates a favorable response of pulpal tissue to CPC than to formocresol. This is in agreement with the findings of earlier studies.^19^ This can be attributed to its biocompatibility, less cytotoxicity and good sealing ability. Moreover, the setting reaction of Chitra- CPC is nonexothermic.^16^ The results of the present study confirm the biocompatibility of CPC, as similar to other studies.^4,16^ The impact of the pulpotomy agent on the pulp was not a discriminating factor because none of the samples in both groups had the same. Dentin chips were present in 60 to 70% of samples in both groups. They promote healing if confined to the superficial portions of the pulp. However, if they are numerous and localized deeper in the pulp tissue they may have a deleterious effect.

**Table 9:** **Table 9: **Histopathological scores and observations after 70 days

		*Material*				*Time period 70 ± 5 days*	
	*Pulpal inflammation*		*Tissue reaction*		*Impaction of particles*		*Presence of dentin chips*
Score	0 1 2 3 4		0 1 2 3		1 2		0 1 2 3
Formocresol	1 2 6 1		10		10		3 7
	*Dentin bridge* *formation*		*Location of* *dentin bridge*		*Quality of* *dentin bridge*		*Connective tissue in* *dentin bridge*
Score	0 1 2 3 4		0 1 2 3		0 1 2 3		0 1 2 3 4 5
Formocresol	8 2		8 2		8 2		8 2
Chitra-CPC	4 2 4		4 3 3		4 2 2		4 3 2 1

                       **DENTIN BRIDGE FORMATION**

**Fig. 2A:**
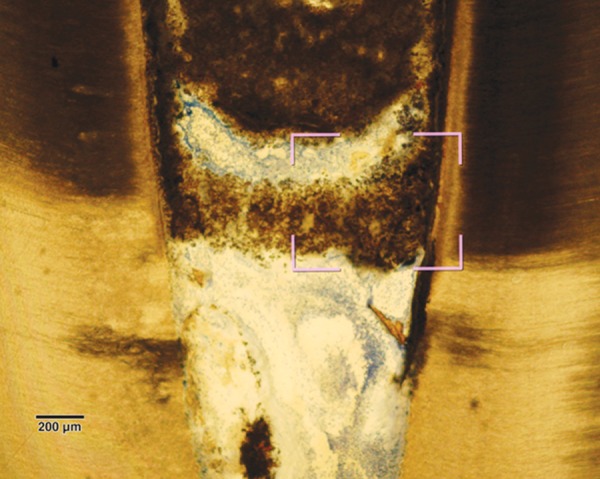
Complete dentin bridge formation in the sample treated with Chitra-CPC (Stevenel's Blue staining, at 4× mag)

**Fig. 2B:**
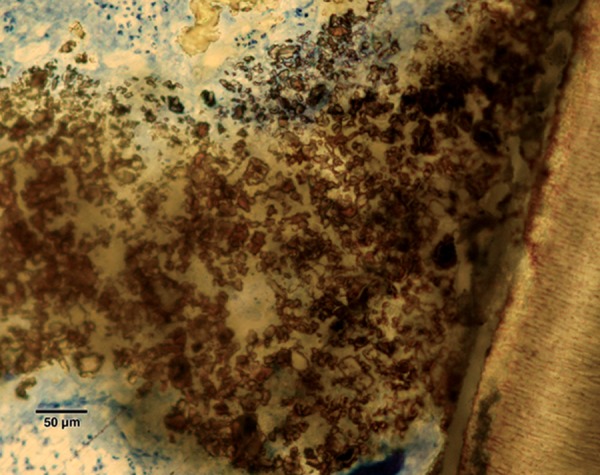
The portion marked is shown magnified (at 20×)

**Fig. 3A:**
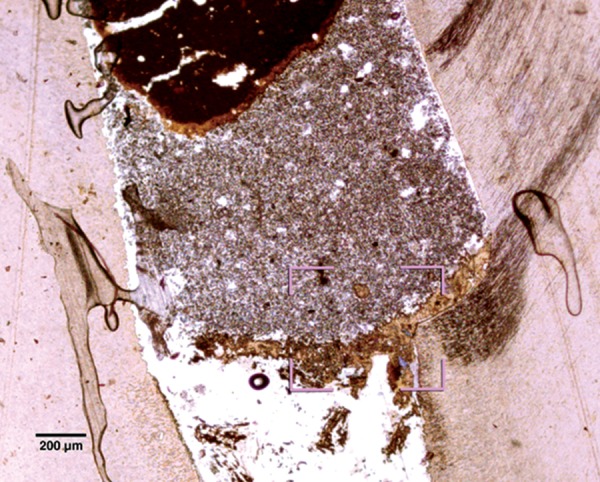
Dentin bridge formation in the sample treated with Chitra-CPC (Stevenel's Blue staining, at 4× mag)

**Fig. 3B:**
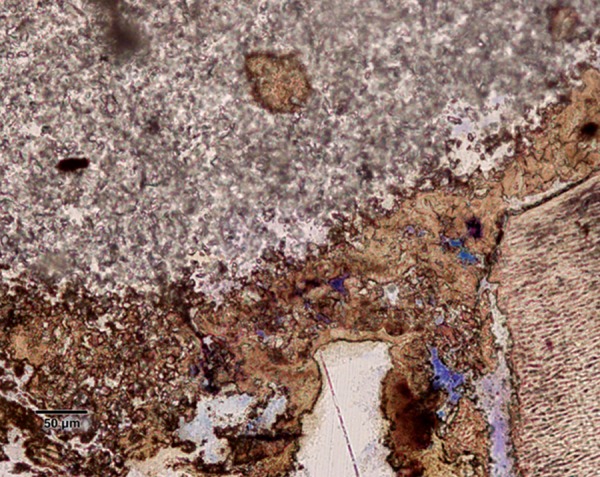
The portion marked is shown magnified (at 20×)

**Fig. 4:**
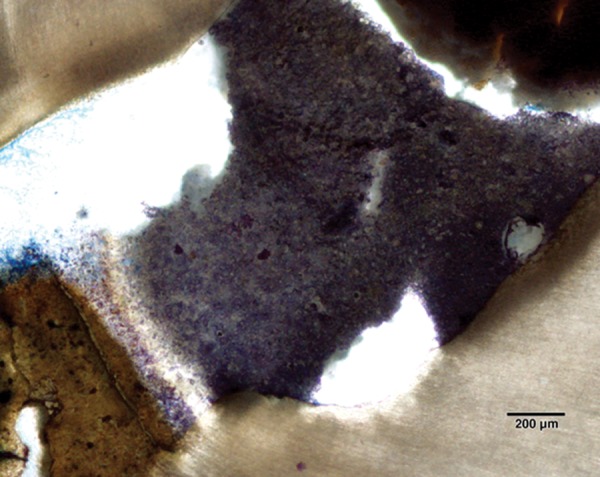
A partial dentine bridge present at the interface with capping agent in the sample treated with Chitra-CPC (Stevenel's Blue staining, at 4× mag)

                       **PULPAL INFLAMMATION**

**Fig. 5A:**
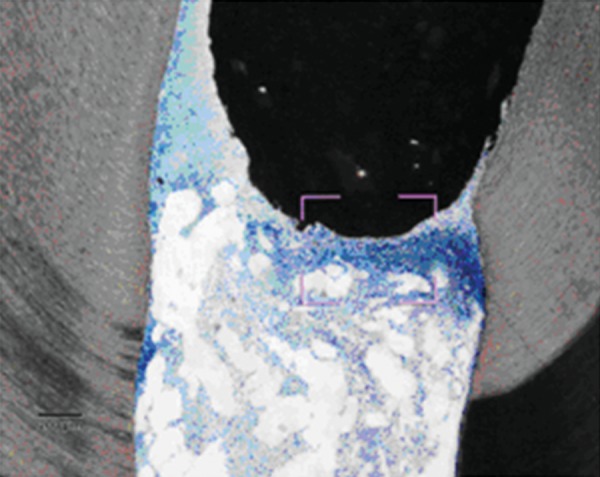
Heavy chronic inflammation in formocresol (Stevenel's Blue staining, at 4× mag)

**Fig. 5B:**
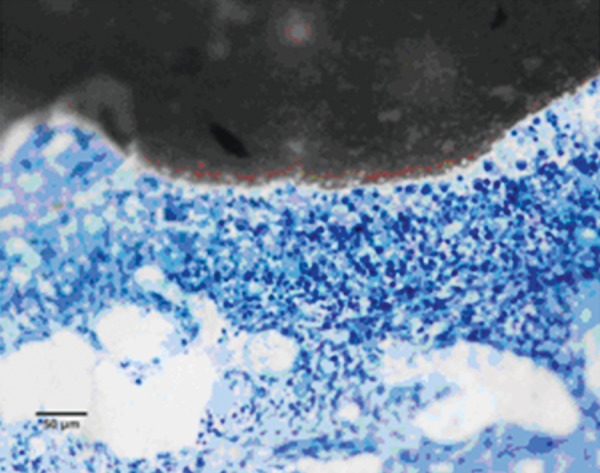
The portion marked is shown magnified (at 20×)

**Fig. 6:**
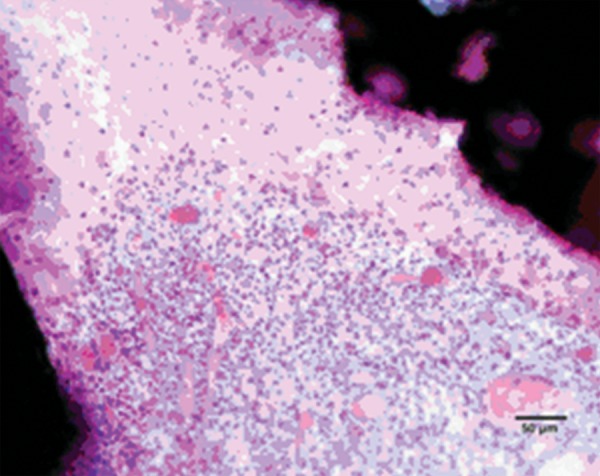
Heavy inflammation is noted in pulp adjacent to sealer in tooth treated with formocresol hematoxylin and eosin staining (at 20×)

**Fig. 7A:**
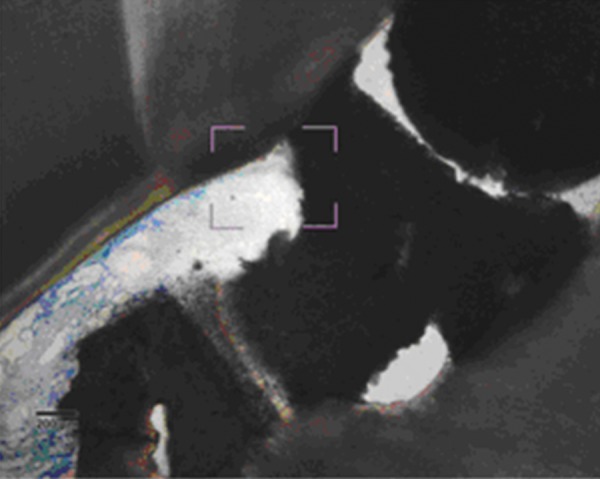
Moderate inflammation in exposed pulp of tooth treated with Chitra-CPC (Stevenel's Blue staining, at 4× mag)

**Fig. 7B:**
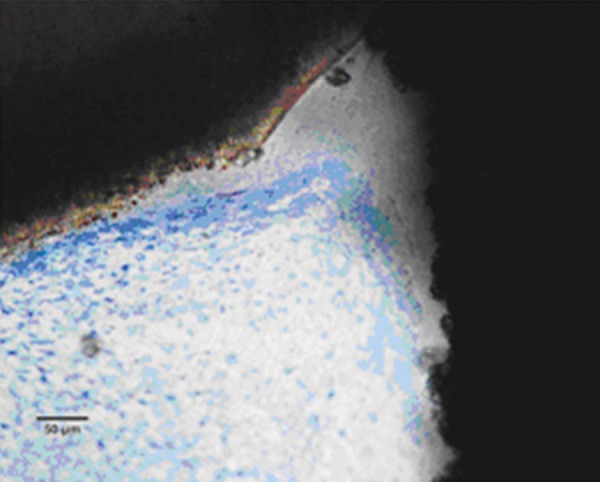
The portion marked is shown magnified (at 20×)

**Fig. 8:**
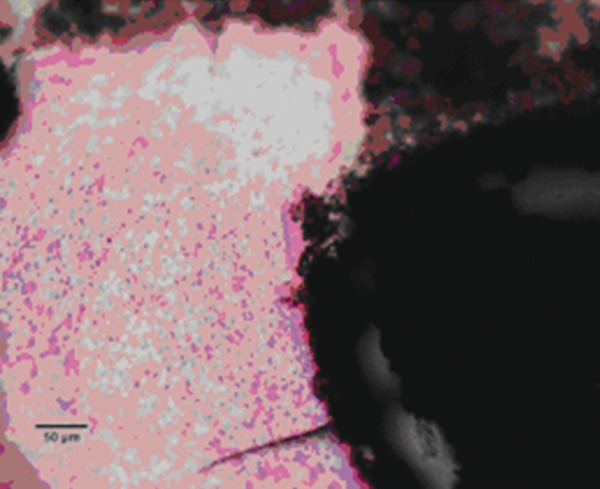
Moderate inflammation is exposed pulp of tooth treated with Chitra-CPC (hematoxylin and eosin staining, at 20×)

The current study showed that CPC promoted a hard tissue formation in the pulp of human deciduous canine teeth. This is in agreement with the findings of earlier studies.^4,19^ Patchy areas of mineralizing reparative dentin were noted along the dentin wall in most of the samples in Chitra-CPC group. It was noted that six samples showed dentin bridge formation. In three samples the dentin bridge formed at the exposure site (Figs 3A to 4) and in three, below the exposure site (Figs 2A and B). In four samples, dentin bridge formation was complete or near complete (Figs 2A to 3B). Partial dentin bridge formation observed in other two samples (Fig. 4). In respect of three samples, regular dentin tubules in the bridge were observed. In most of the samples, dentin bridge did not contain connective tissue. Calcium-phosphate materials induced the formation of dentinal reparatory bridges directly on the biomaterial (Figs 3A to 4), without initial necrosis that occurs inevitably when Ca (OH)_2_ is used.^13^

Theoretically, the biocompatibility of CPC combined with calcium release may allow CPC to stimulate odontoblasts, thus promoting the formation of dentin bridges. The presence of solid substrate matrix, meaning the base on which pulp cells adhere and get transformed into odontoblast like cells, is a necessity for reparatory dentinogenesis.

The ability of CPC to support the formation of a dentin bridge may be attributed to excellent sealing ability^16^ and fast setting. This prevents the diffusion of the material into the tissues, and reduces microleakage during the healing period. Chitra-CPC showed a commendable attachment to dentin walls. The close-pore microstructure, dimensional stability without any shrinkage or crack formation on solidification, and the attachment to dentin walls ensured effective sealing against bacterial microleakage.^16^

Poorly-calcified dentin bridge of connective tissue formed in two samples in formocresol group (Fig. 1). This finding is consistent with various previous studies.^1,6,14^ The presence of a dentin bridge provides natural protection for the pulp against the microleakage of bacteria, and prevents the particles leaching from capping materials from infiltrating into pulp tissues (Stanley, 1998). Furthermore, the formation of a bridge does not imply that the pulp is sealed completely from the environment. The bridge formed is initially permeable but as time progresses, the permeability decreases.

## SUMMARY AND CONCLUSION

The present study showed that Chitra-CPC is biocompatible and it induces initial healing response, replaces with the reparative dentin, creates a scaffold that differentiates cells to attach and secretes mineralized tissue. It, thus, preserves normal histological pulp patterns and pulp vitality in human deciduous teeth. The histological assessment after 70 days revealed no statistically significant difference between the two groups. But Chitra-CPC gave more favorable results with regard to pulpal inflammation, dentin bridge formation, quality of dentin bridge and connective tissue in dentin bridge.

Based on the current study results, it may be concluded that the Chitra-CPC is more biocompatible and it offers an effective healing potential and capability of inducing dentin formation without an area of necrosis. Chitra-CPC, a newer pulp dressing material, which has overcome the disadvantages of the existing ones, would be one of the most desirable pulp dressing agents for deciduous teeth in the coming years. Since the present study, observed only 20 noncarious teeth after 70 days, more experimental data and further human research with larger sample size, longer follow-up period and studies on caries exposed teeth are recommended to conclusively prove the efficacy of Chitra- CPC in inducing a favorable pulpal response.
